# Genetic polymorphisms and population genetic analyses of 57 autosomal InDel loci in Hubei Tujia group

**DOI:** 10.3389/fgene.2023.1066655

**Published:** 2023-03-03

**Authors:** Jiangwei Lan, Xingru Zhang, Wei Cui, Shuyan Mei, Jingtao Xu, Bofeng Zhu

**Affiliations:** ^1^ Guangzhou Key Laboratory of Forensic Multi-Omics for Precision Identification, School of Forensic Medicine, Southern Medical University, Guangzhou, China; ^2^ College of Forensic Medicine, Xi’an Jiaotong University Health Science Center, Xi’an, China

**Keywords:** insertion/deletion polymorphism, East Asian population, Chinese Hubei Tujia group, genetic polymorphism, forensic application, genetic relationship

## Abstract

**Introduction:** The Tujia is the eighth most populous population in China, but its genetic structure has not been fully studied.

**Methods:** In this study, we utilized 57 autosomal Insertion/deletion (InDel) loci to evaluate the genetic polymorphisms and efficiency of forensic applications in the Chinese Hubei Tujia group, and analyzed the genetic structure variances among the studied group and other 26 different reference populations from five continents in 1000 Genomes Project (1KG).

**Results:** The results showed that 57 InDels have no significant deviations from Hardy–Weinberg equilibrium and linkage equilibrium. The combined power of discrimination (CPD) and the combined probability of exclusion (CPE) values for 57 InDels were 0.99999999999999999999999699822 and 0.999975177214539 in the Hubei Tujia group, respectively. In addition, the results of genetic structure analyses indicated that the Hubei Tujia group has close genetic relationships with the Chinese Han population and other East Asian populations.

**Discussion:** These 57 autosomal InDels can be used as reliable tools for forensic individual identification and paternity testing, and are more suitable for East Asian populations. Furthermore, three InDels (rs72085595, rs145941537, and rs34529639) are promising for inferring ancestral information.

## 1 Introduction

Insertion/deletion (InDel) polymorphisms refer to genetic markers with length polymorphisms formed by the insertion or deletion of DNA fragments of different sizes in the genome (Weber et al., 2002). The most widely used genetic marker in DNA database construction and forensic evidence identification so far is short tandem repeat (STR) (Romsos and Vallone, 2015). However, due to the characteristics of long amplified fragment and high mutation rate, the application of STR shows some limitations in difficult and complex cases, especially in degraded sample.

InDels have the advantages of both STR and single-nucleotide polymorphism (SNP) (Sjödin et al., 2010; Mills et al., 2006; Manta et al., 2012; Santos et al., 2010; Romanini et al., 2012), which are widely distributed in the human genome and have a low mutation rate. Compared with STRs, the amplified fragment of InDels are smaller and more suitable for the detection of stale and degraded samples (Jin et al., 2019). InDels are compatible with both high-throughput sequencing platform and capillary electrophoresis platform, which can meet the needs in different forensic DNA laboratories (Tao et al., 2019; Zhang et al., 2020). Therefore, InDels are the genetic markers with great abilities for forensic research and application.

Currently, the Investigator^®^ DIPplex kit (Qiagen, Hilden, Germany) is the most widely used commercial InDel kit. However, it had a relatively low efficacy of forensic application in Chinese populations because the kit was not designed for East Asian or Chinese populations (Bai et al., 2013; Hu et al., 2014; Shen et al., 2016; Cui et al., 2020; Fan et al., 2022). In this study, a newly developed multiplex amplification InDel system, AGCU InDel 60 kit (AGCU ScienTech Incorporation, Wuxi, China), was utilized to evaluate its forensic application value in the Tujia group by analyzing 262 Tujia irrelevant individuals from Hubei province, China. In addition, the genetic polymorphisms of 57 InDels in the Tujia group and the genetic relationships between the Tujia group and other reference populations were further explored with the purpose of enriching the basic genetic data of the Tujia group in China.

## 2 Materials and methods

### 2.1 Sample collection and reference populations

We collected peripheral blood samples or bloodstains from 262 Tujia healthy and unrelated individuals in Hubei province, China, with prior written informed consents, and then stored them at deep cryopreserve or at room temperature in the dry and ventilated place. Genomic DNA of whole blood samples was further extracted using the Magbead Blood Spots DNA kit (CWBIO, Beijing, China) and quantified using the NanoDrop 2000 instrument (Thermo Fisher Scientific, Waltham, MA, United States) following the recommendations of the manufacturers, and then diluted to about 1ng/ul. A total of 26 reference populations from the 1000 Genomes Project phase 3 database were selected as reference populations for population genetic analyses in this study (population information was shown in Supplementary Table S1). These populations were divided into five continental populations, namely, African (AFR), American (AMR), East Asian (EAS), European (EUR), and South Asian (SAS). The research was approved by the Ethics Committee of Xi’an Jiaotong University (No. 2019-1039).

### 2.2 PCR amplification and capillary electrophoresis detection

**FIGURE 1:**
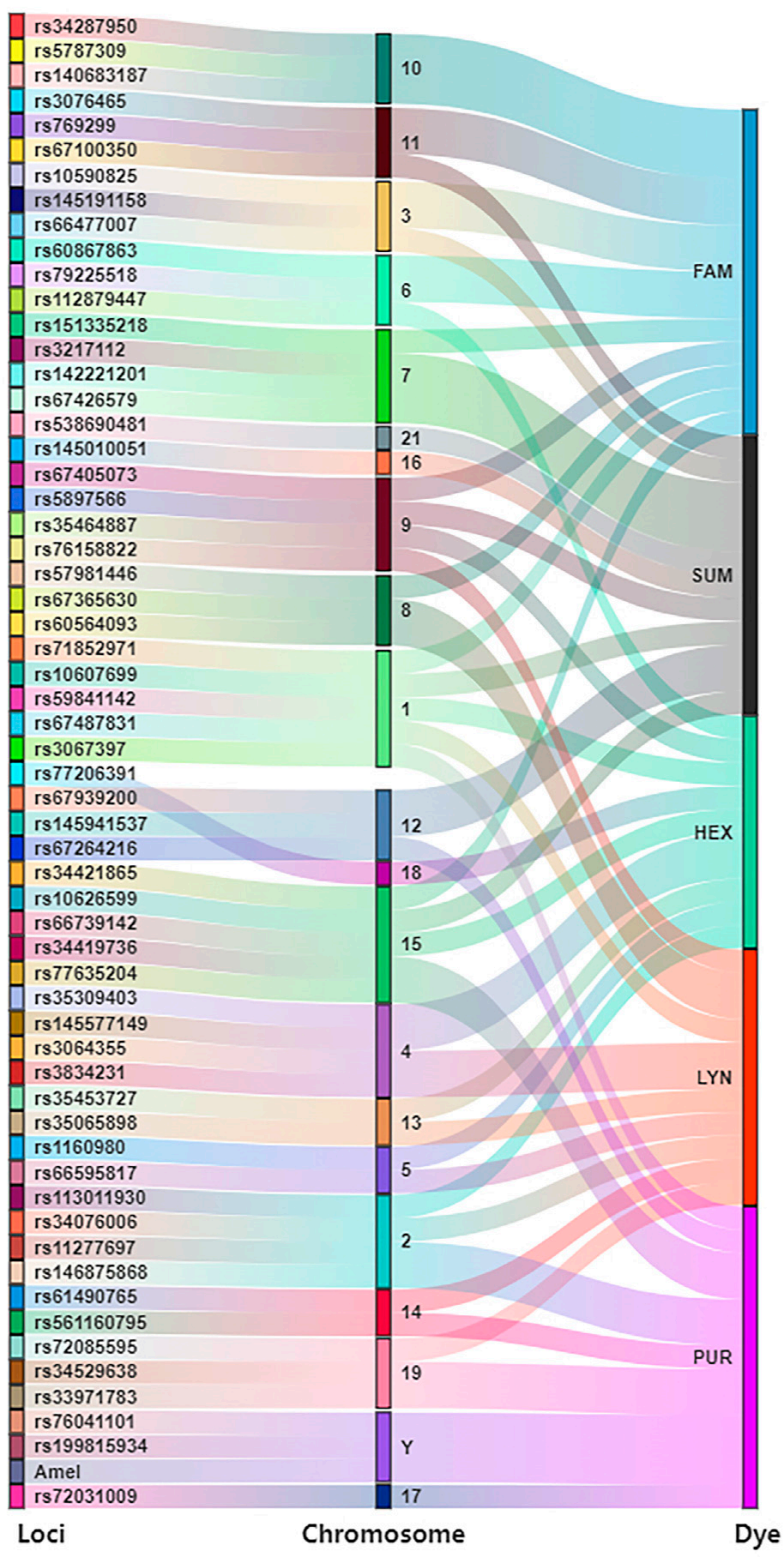
Loci information of AGCU InDel 60 kit.

The AGCU InDel 60 kit is a novel panel with a six-dye multiplex amplification system, which includes 57 autosomal InDels, two Y-chromosomal InDels, and one amelogenin locus. Information (names, chromosomal localizations, and fluorescence labeling) of 60 loci is shown in Figure 1.

Multiplex PCR for 60 InDel loci was performed on the GeneAmp PCR System 9700 Thermal Cycler (Thermo Fisher Scientific, South San Francisco, CA, US) according to the instructions on the AGCU InDel 60 kit. The multiplex PCR system was 25 μL in total which contained 10.0 μL reaction mix, 5.0 μL InDel 60 primers (AGCU ScienTech Incorporation, Wuxi, Jiangsu, China), 1.0 μL U-Taq enzyme, 1.0 ng DNA extracted from whole blood samples or 1mm^2^ bloodstain samples without extraction, and sterilized deionized water. The PCR parameters were as follows: initial denaturation at 95°C for 5 min; then, 28 cycles of denaturation at 94°C for 30s, renaturation at 60°C for 1 min, extension at 62°C for 1 min; and final extension at 72°C for 10 min; and stored at 4°C in the end. After centrifugation at 3000 rpm for 5 min, 1.0 μL amplification products or 1.0 μL InDel 60 Allelic Ladder was mixed with 12.0 μL deionized formamide and 0.5 μL AGCU Marker SIZ-500. The loading mixture was denatured at 95°C for 3 min and then chilled on ice for 3 min immediately. Finally, capillary electrophoresis detection was performed using an ABI 3500xL Genetic Analyzer (Thermo Fisher Scientific, Waltham, Massachusetts, United States), and genotyping was performed using GeneMapper^®^ ID-X Software version 1.5 (Thermo Fisher Scientific, Waltham, Massachusetts, United States). DNA 9948 and deionized water were used as positive and negative controls for each detection.

### 2.3 Statistical analysis

The information of 60 InDels was visualized using the *R* software ggalluvial package. The Hardy–Weinberg equilibrium (HWE) tests of the 57 InDel loci and linkage disequilibrium (LD) analyses of pairwise InDels in the Hubei Tujia group were assessed using STRAF v1.0.5 software (Gou and Alexandre, 2017); in addition, allele frequencies, heterozygosity (Ho), expected heterozygosity (He), polymorphism information content (PIC), discrimination power (PD), probability of paternity exclusion (PE), and probability of match (PM) values were also calculated with this software. Then, the violin map of forensic parameters and the heat map of insertion allele distribution frequencies were drawn using the *R* software ggplot2 package and pheatmap package, respectively. DISPAN program (Ota, 1993) and Arlequin v3.5 software (Excoffier and Lischer, 2010) were used to obtain the values of the pairwise *Nei*’s genetic distances (*D_A_* distances) and fixation index (*F*_*ST*_) among 27 populations. And phylogenetic tree was constructed based on the pairwise *D*_*A*_ distances using the ggtree package (Yu et al., 2018). Principal component analysis (PCA) was performed using *R* software based on population insertion allele frequencies among 27 populations. ADMIXTURE software was used to conduct STRUCTURE cluster analysis for all populations, and the pophelper online tool (http://pophelper.com/) was used to draw the visual bar charts.

## 3 Results

### 3.1 HWE and LD analyses of 57 InDels in the studied Tujia group

The *p*-values for HWE tests at the 57 InDels in the studied Tujia group are shown in Table 1. After the Bonferroni correction, there were no significant deviations from HWE at 57 InDels (*p* > 0.05/57 = 0.00087719). LD tests among these 57 InDels were also evaluated in the studied Tujia group; all pairwise *r* ^*2*^ values were higher than 0.0000313282, indicating that there were no significant LD in these pairs of 57 InDels.

**TABLE 1:** Allelic frequencies and forensic parameters of 57 InDels in the studied Tujia group (*n* = 262) (F_ins_, frequency of the insertion allele; F_del_, frequency of the deletion allele; *P*_HW_, *p*-values for the Hardy–Weinberg equilibrium tests).

Loci	F_ins_	F_del_	He	Ho	PIC	PM	PD	PE	*P*_HW_
rs10590825	0.4924	0.5076	0.5008	0.4656	0.3749	0.3597	0.6403	0.1592	0.2720
rs10607699	0.6489	0.3511	0.4566	0.4198	0.3519	0.3889	0.6111	0.1265	0.2360
rs10626599	0.4733	0.5267	0.4995	0.4580	0.3743	0.3581	0.6419	0.1533	0.2040
rs11277697	0.3798	0.6202	0.4720	0.4695	0.3601	0.3900	0.6100	0.1622	1.0000
rs112879447	0.4447	0.5553	0.4948	0.5000	0.3719	0.3811	0.6189	0.1875	0.9030
rs113011930	0.4561	0.5439	0.4971	0.4847	0.3731	0.3716	0.6284	0.1745	0.6930
rs1160980	0.3855	0.6145	0.4747	0.4275	0.3615	0.3729	0.6271	0.1315	0.1120
rs140683187	0.3569	0.6431	0.4599	0.4466	0.3537	0.3935	0.6065	0.1449	0.6840
rs142221201	0.6603	0.3397	0.4495	0.4885	0.3480	0.4209	0.5791	0.1777	0.1530
rs145010051	0.5038	0.4962	0.5009	0.4504	0.3750	0.3539	0.6461	0.1476	0.1040
rs145191158	0.4561	0.5439	0.4971	0.5000	0.3731	0.3789	0.6211	0.1875	1.0000
rs145577149	0.4027	0.5973	0.4820	0.4924	0.3653	0.3902	0.6098	0.1809	0.7600
rs145941537	0.6355	0.3645	0.4642	0.4313	0.3560	0.3844	0.6156	0.1341	0.2850
rs146875868	0.4160	0.5840	0.4868	0.5115	0.3678	0.3950	0.6050	0.1977	0.4530
rs151335218	0.4599	0.5401	0.4977	0.4771	0.3734	0.3675	0.6325	0.1682	0.5380
rs3064355	0.4294	0.5706	0.4910	0.4466	0.3700	0.3625	0.6375	0.1449	0.1520
rs3067397	0.3149	0.6851	0.4323	0.4466	0.3384	0.4211	0.5789	0.1449	0.6560
rs3076465	0.5095	0.4905	0.5008	0.4542	0.3749	0.3554	0.6446	0.1505	0.1430
rs3217112	0.5725	0.4275	0.4904	0.5191	0.3697	0.3956	0.6044	0.2048	0.3930
rs33971783	0.3931	0.6069	0.4781	0.5038	0.3633	0.3998	0.6002	0.1909	0.4610
rs34076006	0.5076	0.4924	0.5008	0.4962	0.3749	0.3732	0.6268	0.1842	0.9160
rs34287950	0.3779	0.6221	0.4711	0.5115	0.3596	0.4108	0.5892	0.1977	0.1970
rs34419736	0.4198	0.5802	0.4881	0.4656	0.3685	0.3724	0.6276	0.1592	0.5270
rs34421865	0.5878	0.4122	0.4855	0.5038	0.3672	0.3923	0.6077	0.1909	0.6020
rs34529638	0.6469	0.3531	0.4577	0.4466	0.3525	0.3958	0.6042	0.1449	0.7780
rs35065898	0.5706	0.4294	0.4910	0.5076	0.3700	0.3889	0.6111	0.1943	0.6380
rs35309403	0.6221	0.3779	0.4711	0.4656	0.3596	0.3894	0.6106	0.1592	0.9050
rs35453727	0.4046	0.5954	0.4827	0.4809	0.3657	0.3842	0.6158	0.1713	1.0000
rs35464887	0.4523	0.5477	0.4964	0.5076	0.3727	0.3835	0.6165	0.1943	0.8090
rs3834231	0.6393	0.3607	0.4621	0.4924	0.3548	0.4101	0.5899	0.1809	0.3410
rs538690481	0.4237	0.5763	0.4893	0.4504	0.3691	0.3655	0.6345	0.1476	0.2180
rs561160795	0.4160	0.5840	0.4868	0.5573	0.3678	0.4226	0.5774	0.2427	0.0200
rs5787309	0.5382	0.4618	0.4980	0.4733	0.3735	0.3656	0.6344	0.1652	0.4800
rs57981446	0.4828	0.5172	0.5004	0.4771	0.3747	0.3649	0.6351	0.1682	0.4720
rs5897566	0.6355	0.3645	0.4642	0.4389	0.3560	0.3868	0.6132	0.1394	0.4120
rs59841142	0.4637	0.5363	0.4983	0.5305	0.3737	0.3943	0.6057	0.2156	0.2990
rs60564093	0.5840	0.4160	0.4868	0.5267	0.3678	0.4035	0.5965	0.2120	0.2300
rs60867863	0.4771	0.5229	0.4999	0.5267	0.3745	0.3905	0.6095	0.2120	0.4020
rs61490765	0.5248	0.4752	0.4997	0.5076	0.3744	0.3801	0.6199	0.1943	0.8220
rs66477007	0.7328	0.2672	0.3923	0.3435	0.3149	0.4419	0.5581	0.0831	0.0640
rs66595817	0.7290	0.2710	0.3959	0.3664	0.3171	0.4399	0.5601	0.0948	0.2610
rs66739142	0.5210	0.4790	0.5001	0.4771	0.3746	0.3652	0.6348	0.1682	0.4640
rs67100350	0.6393	0.3607	0.4621	0.4695	0.3548	0.3999	0.6001	0.1622	0.8890
rs67264216	0.3760	0.6240	0.4701	0.4771	0.3591	0.3951	0.6049	0.1682	0.8940
rs67365630	0.4943	0.5057	0.5009	0.4695	0.3750	0.3612	0.6388	0.1622	0.3150
rs67405073	0.4237	0.5763	0.4893	0.4656	0.3691	0.3712	0.6288	0.1592	0.4200
rs67426579	0.4637	0.5363	0.4983	0.5076	0.3737	0.3815	0.6185	0.1943	0.7990
rs67487831	0.5630	0.4370	0.4930	0.4847	0.3710	0.3756	0.6244	0.1745	0.7890
rs67939200	0.3760	0.6240	0.4701	0.4618	0.3591	0.3889	0.6111	0.1562	0.8240
rs71852971	0.3206	0.6794	0.4365	0.4351	0.3407	0.4132	0.5868	0.1368	1.0000
rs72031009	0.4637	0.5363	0.4983	0.5076	0.3737	0.3815	0.6185	0.1943	0.8170
rs72085595	0.2901	0.7099	0.4127	0.4122	0.3270	0.4308	0.5692	0.1215	1.0000
rs76158822	0.4828	0.5172	0.5004	0.5382	0.3747	0.3969	0.6031	0.2231	0.2470
rs769299	0.5973	0.4027	0.4820	0.4618	0.3653	0.3770	0.6230	0.1562	0.5430
rs77206391	0.4847	0.5153	0.5005	0.4962	0.3748	0.3736	0.6264	0.1842	0.8860
rs77635204	0.5191	0.4809	0.5002	0.5191	0.3746	0.3858	0.6142	0.2048	0.6210
rs79225518	0.4160	0.5840	0.4868	0.4656	0.3678	0.3737	0.6263	0.1592	0.5180

### 3.2 Allelic frequencies and forensic parameters of 57 InDels in the studied Tujia group

As shown in Table 1, the insertion allele frequencies of the 57 InDels in the studied Tujia group ranged from 0.2901 (rs72085595) to 0.7328 (rs66477007). In order to evaluate the forensic application efficiency of the system of 57 InDels in the studied Tujia group, we calculated the forensic parameters (Ho, He, PIC, PD, PE, and PM). The lowest Ho and He values shown at the locus rs66477007 were 0.3435 and 0.3923, respectively. The rs561160795 locus showed the highest Ho value (0.5573); the rs145010051 and rs67365630 loci showed the highest He value (0.5009). The PIC values of the 57 InDels in the studied Tujia group ranged from 0.3149 (rs66477007) to 0.3750 (rs145010051 and rs67365630). Moreover, the PD, PE, and PM values of the 57 InDels varied from 0.5581 (rs66477007) to 0.6461 (rs145010051), 0.0831(rs66477007) to 0.2427 (rs561160795), and 0.3539 (rs145010051) to 0.4419 (rs66477007) in the studied Tujia group, respectively. The values of combined PD (CPD) and combined PE (CPE) were calculated among the joint application of the 57 InDels, which reached to 0.99999999999999999999999699822 and 0.999975177214539, respectively.

### 3.3 Forensic efficiency comparisons of 57 InDels between the studied Tujia group and other 26 reference populations

**FIGURE 2:**
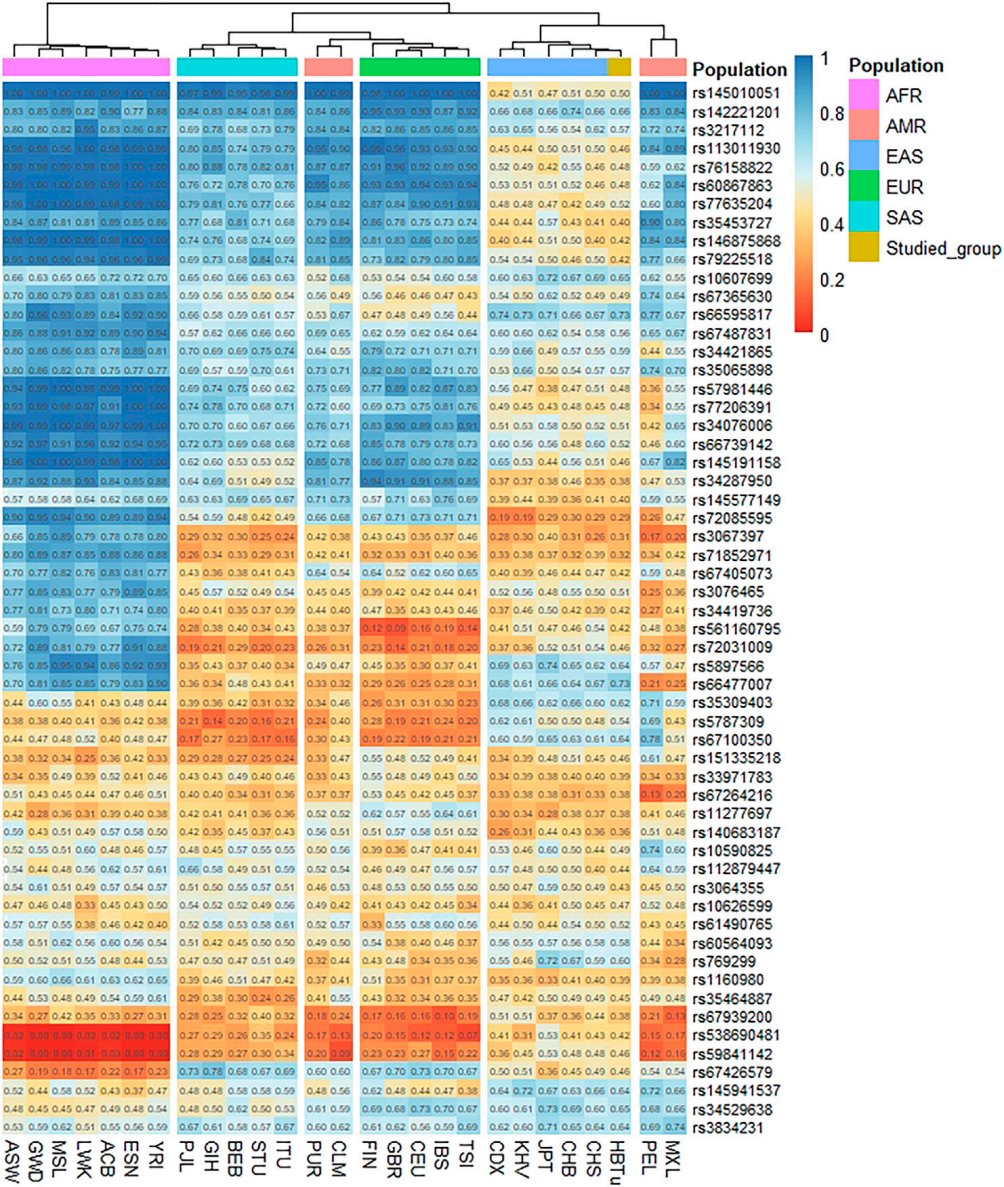
Heatmap showing the insertion allele frequency differences among the studied Tujia group and 26 reference populations on the same 57 autosomal InDels.

The heatmap based on the insertion allele frequencies of 57 InDels for the studied Tujia group and 26 reference populations is shown in Figure 2. The frequency values were expressed by different color of the cube in the heat map; the color changed from blue to orange as the insertion allele frequency values decreased. As shown in Figure 2, the allele frequency distributions of the studied Tujia group were similar to those of the five reference populations in EAS and showed significant differences from those of other four reference intercontinental populations.

Cluster analysis showed that these 27 populations were mainly divided into two clusters, and seven AFR populations constituted one of the main clusters. As shown in Figure 2, twelve InDels (loci rs145010051, rs113011930, rs76158822, rs60867863, rs77635204, rs46875868, rs79225518, rs57981446, rs77206391, rs34076006, rs66739142, and rs145191158) were high insertion allele frequency distributions (>0.9) in AFR populations. The other main cluster was composed of four continental populations: EUR, SAS, EAS, and AMR. The studied Tujia group in this research was in the same sub-cluster with EAS populations due to their relatively similar insertion allele frequency distributions. Except for loci rs72085595, rs3067397, rs71852971, rs67264216, and rs11277697, the insertion allele frequencies of other loci ranged from 0.4 to 0.7 in EAS populations. In addition, we also found that some loci showed particular allele frequency distributions in different continental populations. For example, the insertion allele frequency of the locus rs72085595 was lower in the studied Tujia group and EAS populations but higher in the AFR and EUR populations. Meanwhile, loci rs145941537 and rs34529639 were higher frequency values in the studied Tujia group and EAS populations but lower frequency values in the AFR and EUR populations.

**FIGURE 3:**
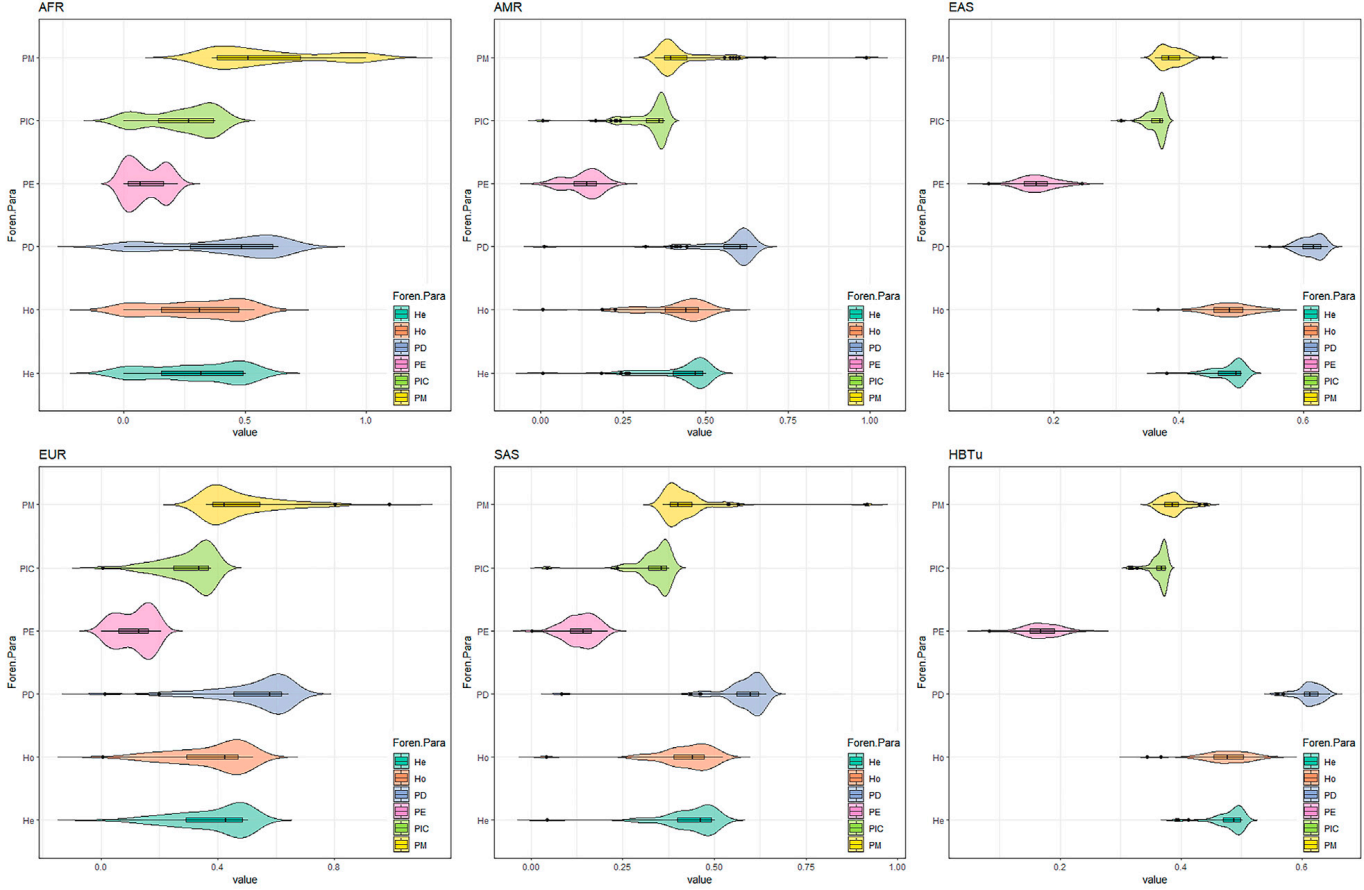
Forensic parameters (PM, PIC, PE, PD, Ho, and He) comparisons of 57 InDels between the studied Tujia (HBTu) group and five intercontinental populations.

Based on these 57 InDels, we compared the relevant forensic parameters (He, Ho, PD, PE, PIC, and PM) of the studied Tujia group and five continental populations. As shown in Figure 3, the PIC values of 57 InDel loci in the studied Tujia group and five continental populations ranged from 0.001511 to 0.374979 (AFR), 0.005731 to 0.375 (AMR), 0.307745 to 0.374965 (EAS), 0.005929 to 0.374937 (EUR), and 0.043011 to 0.374983 (SAS). The He values ranged from 0.001513 to 0.500337 (AFR), 0.005755 to 0.500722 (AMR), 0.380288 to 0.500426 (EAS), 0.005952 to 0.500371 (EUR), and 0.044023 to 0.500478 (SAS). The Ho values varied from 0.001513 to 0.537065 (AFR), 0.005764 to 0.544669 (AMR), 0.367063 to 0.559524 (EAS), 0.005964 to 0.518887 (EUR), and 0.0409 to 0.521472 (SAS). The PD values varied from 0.003021 to 0.636413 (AFR), 0.011461 to 0.65417 (AMR), 0.544871 to 0.638314 (EAS), 0.011857 to 0.641076 (EUR), and 0.082368 to 0.640437 (SAS). The PM values ranged from 0.363587 to 0.996979 (AFR), 0.34583 to 0.988539 (AMR), 0.361686 to 0.455129 (EAS), 0.358924 to 0.988143 (EUR), and 0.359563 to 0.917632 (SAS). The PE values ranged from 0.000002282 to 0.222042 (AFR), 0.0000328 to 0.229663 (AMR), 0.09511 to 0.245095 (EAS), 0.00003515 to 0.20456751 (EUR), and 0.001547 to 0.20699 (SAS).

### 3.4 Population genetic relationship analyses between the studied Tujia group and 26 reference populations

**FIGURE 4:**
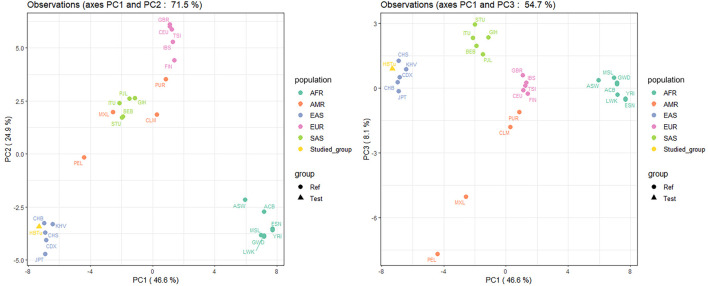
Principal component analyses (PCAs) of the studied Tujia group and 26 reference populations based on 57 autosomal InDels at the population level.

We evaluated the population genetic relationships of the studied Tujia group and 26 reference populations by PCA, structure, *D*_*A*_, and *F*_*ST*_ analyses. As shown in Figure 4, we utilized two PCA plots to illustrate the genetic relationships among these 27 populations based on 57 InDels at the population level, where the same color represented the same continental populations, however, the studied Tujia group was colored in separate color. The first, second, and third components (PC1, PC2, and PC3) could explain 46.6%, 24.9%, and 8.1% of the total variance, respectively. The AFR and EAS populations could be distinguished from other continental populations on PC1, the EUR populations on PC2, and the SAS populations on PC3. The studied Tujia group was located at the cluster of EAS populations.

**FIGURE 5:**
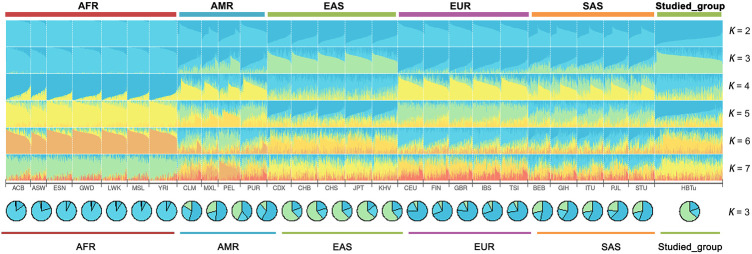
Population STRUCTURE analyses among the studied Tujia group and 26 reference populations based on the same 57 autosomal InDels using ADMIXTURE software at *K* = 2–7.

STRUCTURE analyses of the studied Tujia group and 26 reference populations at the individual level are shown in Figure 5. At *K* = 2, the ancestral components of AFR populations were mainly light blue, while those of EAS populations were mainly dark blue. At *K* = 3, the ancestral components of AFR populations were mainly light blue, those of EAS populations were mainly green, and those of EUR populations were mainly dark blue. With the increase in the *K* values, the AMR and SAS populations also gradually showed different ancestral compositions compared with AFR, EUR and EAS populations. Regardless of the values of *K*, the studied Tujia group always showed similar ancestral compositions with the EAS populations.

**FIGURE 6:**
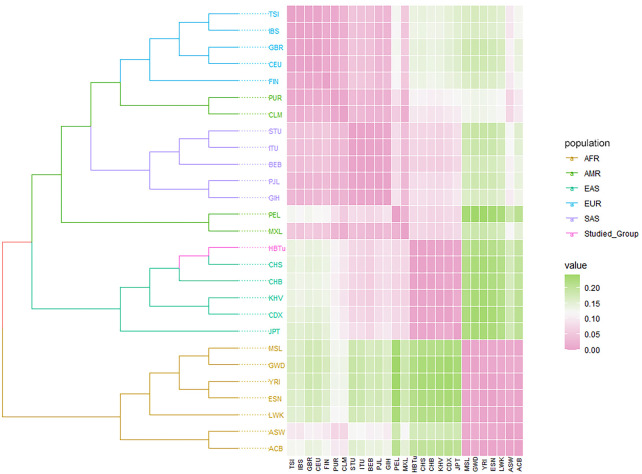
Phylogenetic tree based on pairwise *D*_*A*_ genetic distances, and *F*_*ST*_ heatmap of the studied Tujia group and 26 reference populations.

The phylogenetic tree on the basis of pairwise *D*_*A*_ genetic distances, and *F*_*ST*_ heatmap could visualize the genetic relationships of the studied Tujia group and 26 reference populations. As shown in Figure 6, these 27 populations were divided into two main branches: one was composed of seven AFR populations, while the other consisted of EAS (including the studied Tujia group), SAS, AMR, and EUR populations. Specifically, the studied Tujia group and CHS were clustered firstly, and then incorporated into the CHB and other three EAS populations in turn; all six EAS populations gathered into one branch eventually. The phylogenetic tree and heat map revealed that the genetic relationships between the studied Tujia group and EAS populations were closer than those between the studied Tujia group and AFR populations, correspondingly.

## 4 Discussion

The Tujia group is one of the 56 populations in the People’s Republic of China, mainly distributed in the Wuling Mountain area at the junction of Hunan, Hubei, Chongqing and Guizhou provinces (Compilation group of a Brief History of Tujia, 2009). According to the statistics (National Bureau of Statistics, 2021), the population of the Tujia nationality in China is 9,587,732 (until 31 December 2020), ranking eighth among all populations in China.

In this study, an InDel kit was used to analyze the genetic diversities of 57 autosomal InDels in 262 unrelated Tujia individuals living in Hubei province, China. The pairwise LD and HWE analyses of 57 InDels indicated that these loci could be used for CPD and CPE calculations in Tujia group and population genetic structure analyses. In order to evaluate the forensic application efficiencies of 57 InDels in the studied Tujia group, we calculated the forensic parameters of 57 InDels, and the results showed that all 57 InDels were polymorphic (Botstein et al., 1980), and the PIC values of the 57 InDels were all above 0.3. The CPD and CPE values were 0.99999999999999999999999699822 and 0.999975177214539 in the studied Tujia group, respectively, indicating that the panel can meet the requirements of forensic individual identification and paternity testing.

Consistent with the previous studies, the AGCU InDel 60 kit outperformed the Investigator^®^ DIPplex kit for individual identification and paternity testing in East Asians. The current study also confirmed this conclusion by detecting 262 Tujia individuals using the AGCU InDel 60 kit. The CPD and CPE values of the Tujia group calculated by these 57 InDel loci were higher than those values calculated using the Investigator^®^ DIPplex kit (0.99999999761 and 0.9860) in the previous literature (Shen et al., 2016). Furthermore, for the comparisons of forensic parameters in five continental populations by 57 InDels (Figure 3), the genetic polymorphisms of 57 InDel loci in this panel were higher in the studied Tujia group and EAS populations, which indicated that this panel could not only effectively compensate for the shortcoming of the current commercial kit in the individual identification and paternity testing of these six EAS populations but also be used as a valid tool for individual identification and paternity testing in the Hubei Tujia group.

According to the STRUCTURE result (Figure 5), regardless of the values of *K*, the studied Tujia group always showed similar ancestral components to EAS populations. When *K* = 3, the studied Tujia group and EAS, AFR, and EUR populations could be clearly distinguished based on their different ancestral components. However, the inability to distinguish AMR and SAS populations might be related to the lack of sufficient ancestral information in this panel. As shown in the heat map of the insertion allele frequencies (Figure 2), the insertion allele frequencies of some loci (rs72085595, rs145941537, and rs34529639) were distinctly different among some continental populations. These results indicated that these three loci were also valuable for inferring ancestral information among the three continental populations of EAS, AFR, and EUR. These loci which showed significant variations in the allele frequencies among different populations were referred to as ancestry informative markers.

Using the comparisons of insertion allele frequencies for the studied Tujia group and 26 reference populations in 1KG (Figure 2), we observed that there were more similar allele frequency distributions between populations within the same continent, yet obvious differences among various continents, especially among EAS, AFR and EUR populations. Moreover, the studied Tujia group showed relatively close genetic relationships with five reference EAS populations. Based on 57 InDels, we calculated pairwise *Nei*’s *D*_*A*_ and *F*_*ST*_ values and took advantage of PCA and STRUCTURE genetic analysis methods to evaluate the population genetic relationships between the studied Tujia group and 26 reference populations in the five continents. PCA can extract principal components from complex multivariate data and intuitively show the genetic relationships between various populations through the near and far relative positions of different populations. As shown in Figure 4, PC1, PC2, and PC3 could explain 79.6% of the total variation. The studied Tujia group and EAS populations clustered together in PCA plots, indicating that the studied Tujia group has close genetic relationships with EAS populations. The phylogenetic tree and heatmap (Figure 6) based on *D*_*A*_ and *F*_*ST*_ values also confirmed the aforementioned viewpoint; in the EAS branch, the studied Tujia group and CHS population clustered first, then incorporated into the CHB population, and finally, the KHV, CDX, and JPT populations gathered in the branch. In addition, the *F*_*ST*_ values between the studied Tujia group and EAS populations were smaller than those of other continental populations. These results indicated that there were close genetic relationships between the studied Tujia group, the Chinese Han populations, and EAS populations. In particular, the Tujia group was closely related to the CHS population at the genetic level.

As an ethnic group with a long history, the Tujia has its own national language Bifzivkar, without characters, which belongs to the Tibeto-Burman language family of the Sino-Tibetan languages and is close to the Yi language. The genetic origin of the Tujia is still inconclusive in academia (Yang R, 2005; LI, 2013; Wang et al., 2018). With the expansion of Han population in the Song Dynasty, the social customs of the Han began to affect the Tujia group (Duan, 2000). Since the implementation of bureaucratization of native officers in the fifth year of Huuwaliyasun Tob in the Qing Dynasty (1727 A.D.), a large number of Han people had migrated to Southwest China, and the civil exchanges between the Tujia and Han population had become more frequent. Long-term cultural interaction and intermarriage had promoted ethnic integration (Li and Wang, 2012). The results revealed in this study are consistent with the the development history of the Tujia group.

## 5 Conclusion

In this study, 60 InDels from the AGCU InDel 60 kit were detected on 262 Hubei Tujia individuals using the capillary electrophoresis platform, and the genotyping results of 57 autosomal InDels were analyzed for revealing forensic efficiency. The high CPD and CPE values obtained in this study revealed that this AGCU InDel 60 kit can effectively compensate the deficiency of other commercial kits used in EAS populations and can be used as an effective tool for individual identification and parentage testing in the Hubei Tujia group. Moreover, the results of population genetic analyses indicated that the studied Tujia group has a closer genetic relationship with the CHS population.

## Data Availability

The datasets for this article are not publicly available due to concerns regarding participant/patient anonymity. Requests to access the datasets should be directed to the corresponding authors.
